# Supplementary material for: A novel lipase with dual localisation in Trypanosoma brucei
Source: Sci Rep. 2022 Mar 19;12:4766. doi: 10.1038/s41598-022-08546-w (PMC8934347; doi:10.1038/s41598-022-08546-w)
Supplement: Supplementary file 1 — Supplementary Information 1. [file 41598_2022_8546_MOESM1_ESM.pdf]

A

B

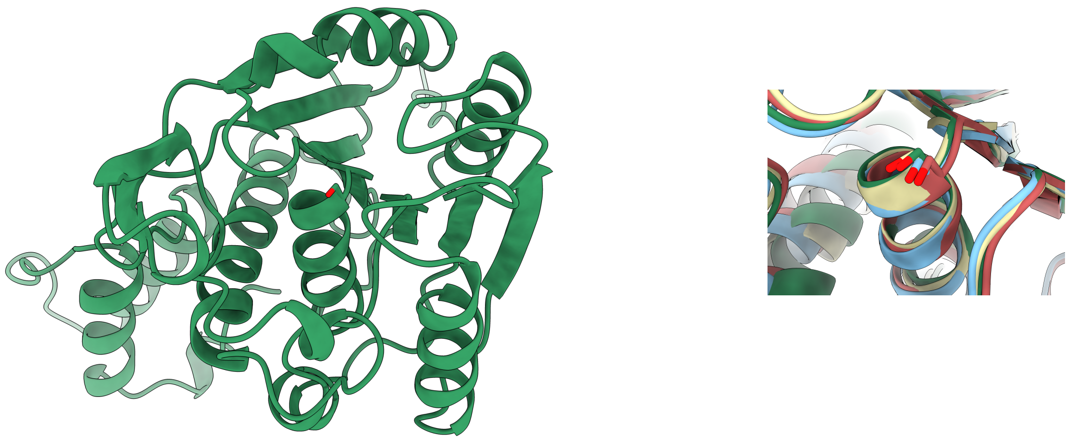

Fig S1

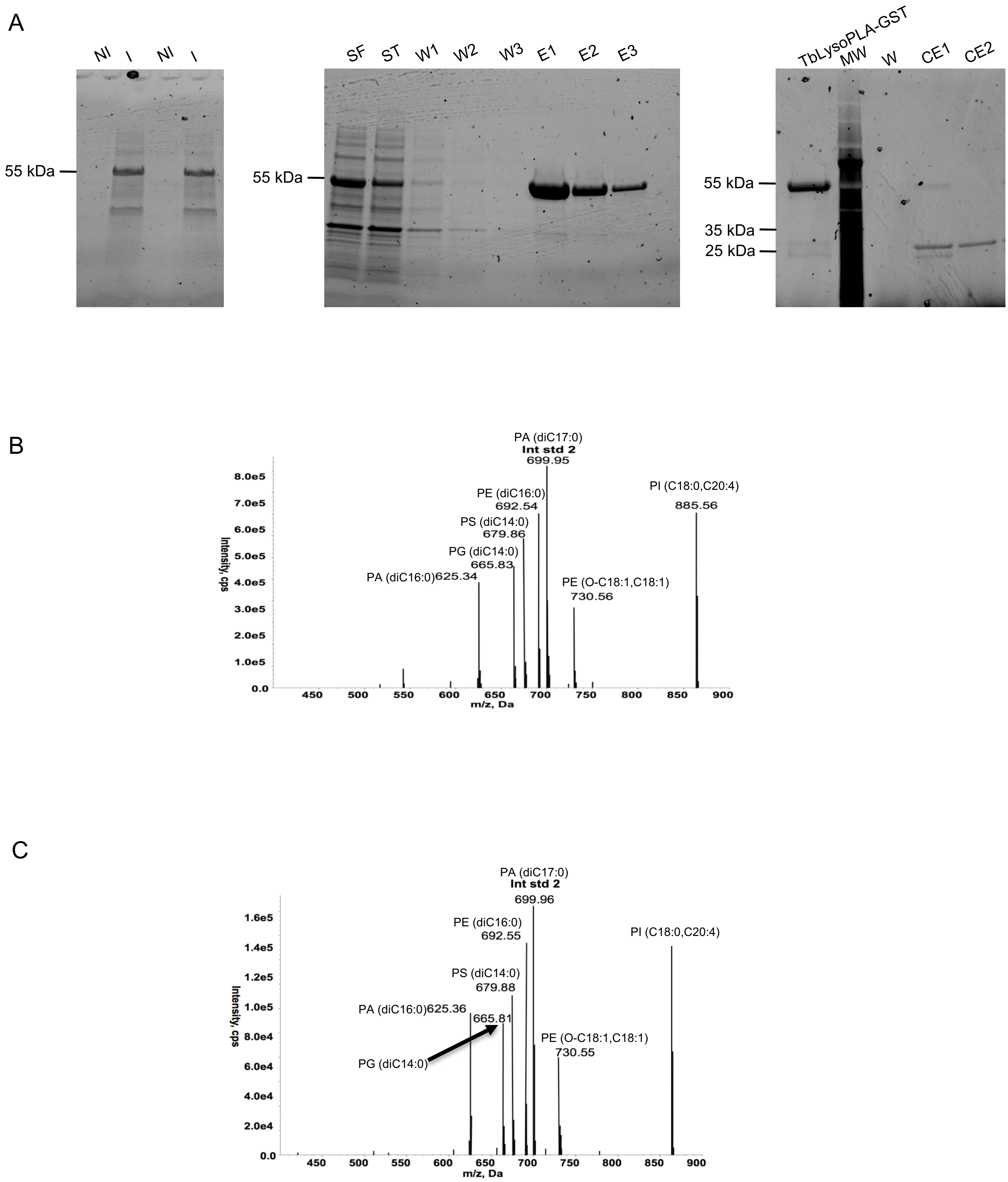
A
NI
I
NI
I
55 kDa
SF
ST
W1
W2
W3
E1
E2
E3
55 kDa
TbLysoPLA-GST
MW
W
CE1
CE2
55 kDa
35 kDa
25 kDa
B
PA (diC17:0)
Int std 2
699.95
PE (diC16:0)
692.54
PS (diC14:0)
679.86
PG (diC14:0)
665.83
PA (diC16:0)625.34
PE (O-C18:1,C18:1)
730.56
PI (C18:0,C20:4)
885.56
Intensity, cps
m/z, Da
C
PA (diC17:0)
Int std 2
699.96
PE (diC16:0)
692.55
PS (diC14:0)
679.88
PA (diC16:0)625.36
665.81
PG (diC14:0)
PE (O-C18:1,C18:1)
730.55
PI (C18:0,C20:4)
Intensity, cps
m/z, Da

Fig S2

A

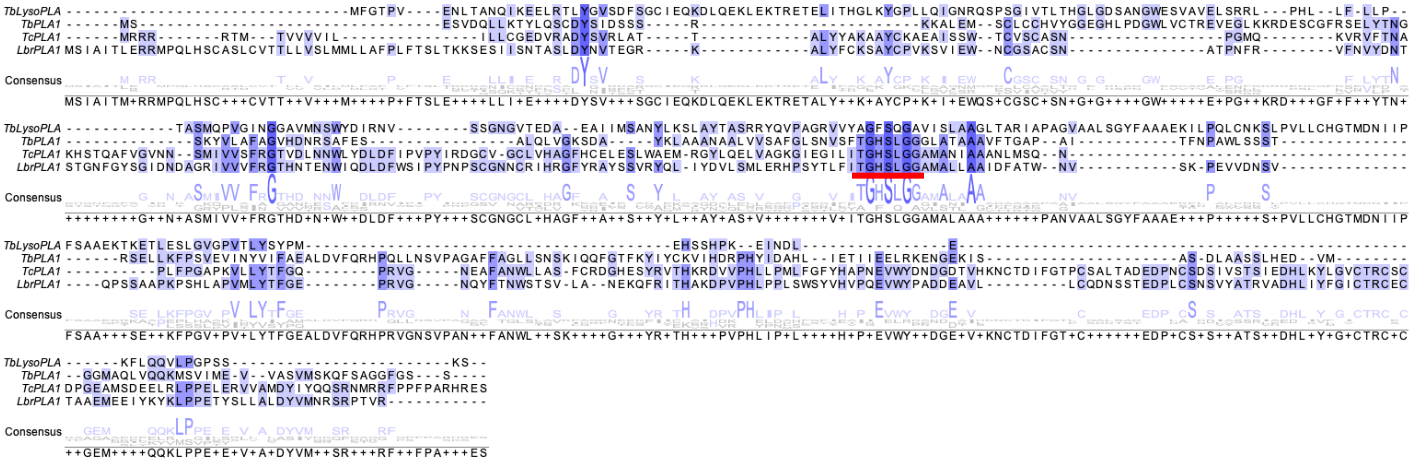

TbLysoPLA
TbPLA1
TcPLA1
LbrPLA1
Consensus

B

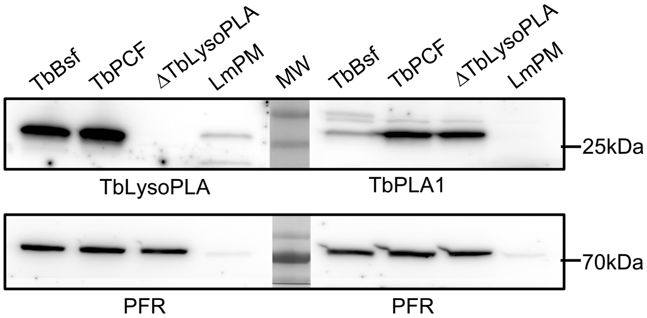

TbBsf
TbPCF
ΔTbLysoPLA
LmPM
MW
TbBsf
TbPCF
ΔTbLysoPLA
LmPM
25kDa
TbLysoPLA
TbPLA1
70kDa
PFR
PFR

Fig S3

A

B

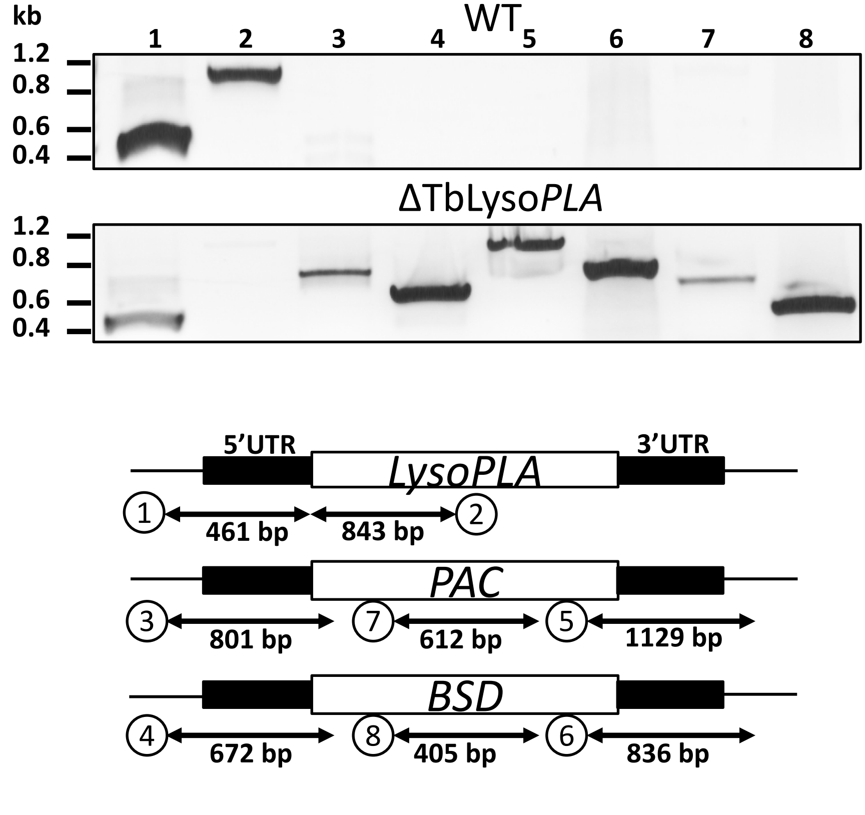

kb
WT
1
2
3
4
5
6
7
8
1.2
0.8
0.6
0.4
ΔTbLysoPLA
5'UTR
3'UTR
LysoPLA
461 bp
843 bp
PAC
801 bp
612 bp
1129 bp
BSD
672 bp
405 bp
836 bp

Fig S4

	TbLysoPLA	TbgLysoPLA	TevLysoPLA	TcoLysoPLA	TviLysoPLA	TcrLysoPLA	LmLysoPLA
TbLysoPLA	100						
TbgLysoPLA	99.64	100					
TevLysoPLA	99.64	99.64	100				
TcoLysoPLA	70.61	70.61	70.61	100			
TviLysoPLA	52.17	52.17	52.17	51.19	100		
TcrLysoPLA	62.86	62.50	62.50	63.44	54.33	100	
LmLysoPLA	53.82	53.82	53.82	53.28	43.60	57.61	100

Table S1

	TbLysoPLA	TbPLA1a	TcrPLA1	LbrPLA
TblysoPLA	100			
TbPLA1a	16.06	100		
TcrPLA1	16.29	20.83	100	
LbrPLA	18.27	17.25	37.50	100

Table S2

Name	Sequence	Purpose
PLA1bpGEXfw	AG**CTCGAG**ATGTTTGGCACGCCGGTTGAGAAT	Heterologous expression in E.coli
PLA1bpGEXfw	AG**GCGGCCGC**TTACGATTTCGATGAAGGTCCGGG	
PLA1bMDfw	TGCCGGATTC*G*CGCAAGGTGC	
PLA1bMDrev	GCACCTTGCG*C*GAATCCGGCA	
PLA1bRNAisens5p	GC**AAGCTT**ATGTTTGGCACGCCGGTTGA	Interference RNA
PLA1bRNAisens3p	GC**CTCGAG**TACTCCATTCCCTGAGCTCA	
PLA1bRNAiantisens5p	GC**AAGCTT**GAA**CTCGAG**AGACTTTTGAGGTAATTGGC	
PLA1bRNAiantisens3p	GC**TCTAGA**ATGTTTGGCACGCCGGTTGA	
PLA1b5UTRfw	GTTGTG**CCATGG**GTGTCC**ATTAAC**TCAACTAT	Knock-out
PLA1b5UTRrev	CAACCG**GAATTC**CAAACAT**TCTAGA**CAACTTTCAGTGAGGTTTCGTAAATG	
PLA1b3UTRfw	CCTTCA**TCTAGA**TCGTAA**CTCGAG**TGGAGTTGTAAGAAAGAGAGGTGAATG	
PLA1b3UTRrev	AACGGC**GCCTTA**TAAT**GCAAAC**GTGTTTTGGTGCTTGCTCCTACGGC	
PLA1bTyNterfw	GC**AAGCTT**ATGGAGGTCCATACTAACCAAGATCCACTTGACGCCTTTGGCACGCCGGTTGAGAAT	Expression in Trypanosomes
PLA1bTyNterrev	GC**CTCGAG**TTACGATTTCGATGAAGGTCCGGG	
PLA1bnoSKSrev	GC**CTCGAG**TTATGAAGGTCCGGGGAGGACTTGCT	
PLA1bSKLrev	GC**CTCGAG**TTACAATTTCGATGAAGGTC	

In **bold**: restriction sites for cloning
Underlined: sequence TY tag
Italic: change to create a mutation

Table S3
