# Supplementary material for: A novel lipase with dual localisation in Trypanosoma brucei
Source: Sci Rep. 2022 Mar 19;12:4766. doi: 10.1038/s41598-022-08546-w (PMC8934347; doi:10.1038/s41598-022-08546-w)
Supplement: Supplementary file 2 — Supplementary Information 2. [file 41598_2022_8546_MOESM2_ESM.docx]

# Supplemental

**Figure S1: Structure prediction of LysoPLA using Alpha-Fold**

A/ Prediction of TcLysoPLA using Alpha-fold. B/ Alignment of predicted catalytic site

Pale cyan = Rhodobacter sphaeroides esterase (RspE) pdb id = 4FHZ; Pale Yellow = Francisella tularensis carboxylesterase (FTT258) pdb id = 4F21; Salmon = Human LYPLAL1= pdb id 3U0V; Pale blue = Human lysophospholipase A2 (LYPLA2) pdb = 6BJE.

**Figure S2: Expression and purification of TbLysoPLA. A/** Left: Expression in *E. coli* BL21Star, non-induced, NI; induced with IPTG, I. Middle: Purification steps using Glutathion sepharose beads followed by glutathione elutions. SF (Soluble Fraction), FT (Flow Through), W1-2 (Washs), E1-3 (elutions with glutathion). Right: Thrombin clivage. MW, Molecular weight; W (Wash); CE1-2 (Clivage-Elution).

B/ Substrate specificity of recombinant TbLysoPLA. TbLysoPLA was incubated (B) or not (C) with a Lipid Mix containing PA (diC16:0), PA (diC17:0) PG (diC14:0), PE (O-C18:1, C18:1), PE (diC16:0), PS (diC14:0), PI (C18:0, C20:4).

**Figure S3: Comparison of TbLysoPLA and TbPLA1.** A/ Multiple sequence alignment of TbLysoPLA and Tb, Tcr and Lbr PLA1. Amino acid sequences were aligned with Clustal Omega using basic settings and edited using the Jalview software. Dark blue contains conserved residues, white to light blue contains conservative changes. The lipase consensus pattern is underscored by a red lign. TbLysoPLA, *Trypanosoma brucei* LysoPLA (Accession number in GeneDB Tb927.8.6390); TbPLA1, *Trypanosoma brucei* PLA1 (Accession number CAG29794, ^19^); TcPLA1, *Trypanosoma cruzi* PLA1 (Accession number JN975637, ^13^); LbrPLA1, *Leishmania braziliensis* PLA1 (Accession number KJ957826, ^14^).

B/ Western Blot analysis using anti-TbLysoPLA and anti TbPLA1b antibodies. Total protein extracts were resolved by SDS-PAGE and transferred on nitrocellulose as described in materiel and method section. Membranes were probed with anti-TbLysoPLA, anti-TbPLA1and anti-PFR. TbBsf, *T. brucei* Bloodsream form; TbPCF, *T. brucei* Procyclic form; TbLysoPLA; TbLysoPLA knock-out cell-line Tb bloodstream form; LmxPM, *Leishmania Mexicana* promastigote form. PFR was used as loading control.

**Figure S4: Generation of the ∆TbLysoPLA cell-line**. TbLysoPLA alleles were replaced in the Tb427 parental cell-line by blasticidin and puromycin resistance genes.

A / Representation of the wild type and recombinant loci and the PCR strategy to identify marker integration and wild-type loci.

B / Analysis of genomic DNA extraction from parental cell-line (WT) or from a clone ∆TbLysoPLA by PCR amplification of the fragments presented in A (for primers see supplementary table).

**Table S1**: LysoPLA: percentage of identity among kinetoplastids. Protein sequences were aligned using Clustal Omega Algorythm with basic settings then identity matrix was retrieved.

**Table S2**: **Comparison of TbLysoPLA and TbPLA1**. Protein sequences were aligned using Clustal Omega Algorythm with basic settings then identity matrix was retrieved.

**Table S3: Primers used for PCR**
